# Supplementary material for: Sex-biased admixture and assortative mating shape genetic variation and influence demographic inference in admixed Cabo Verdeans
Source: G3 (Bethesda). 2022 Jul 21;12(10):jkac183. doi: 10.1093/g3journal/jkac183 (PMC9526050; doi:10.1093/g3journal/jkac183)
Supplement: jkac183_Supplementary_Fig_1 [file jkac183_supplementary_fig_1.pdf]

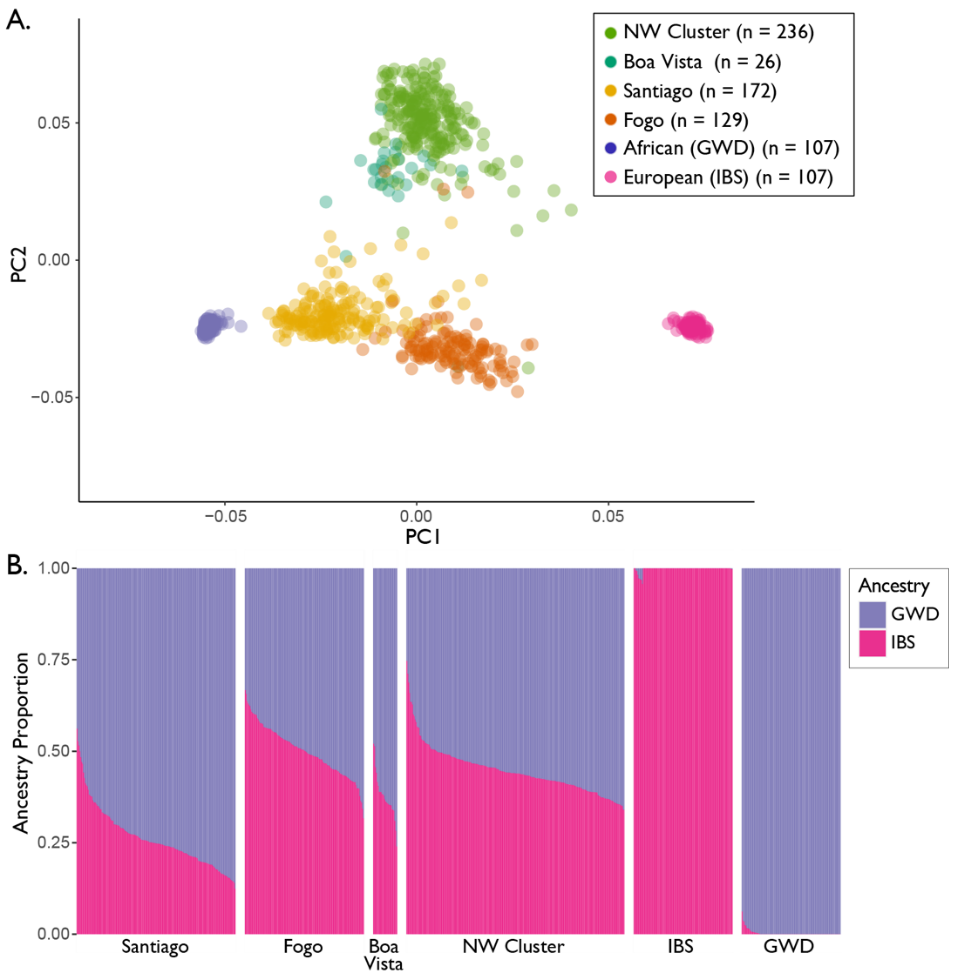

Supp Fig 1: Principal Components Analysis and estimated admixture proportions. (A) The first two PCs are shown, based on the pruned autosomal dataset of 514,551 SNPs from Cabo Verdean individuals (colored based their island of origin) and reference populations (West African and European), with sample sizes noted in the legend. (B) ADMIXTURE estimates of overall autosomal ancestry per individual, grouped by island region or reference population.
