# Supplementary material for: Sex-biased admixture and assortative mating shape genetic variation and influence demographic inference in admixed Cabo Verdeans
Source: G3 (Bethesda). 2022 Jul 21;12(10):jkac183. doi: 10.1093/g3journal/jkac183 (PMC9526050; doi:10.1093/g3journal/jkac183)
Supplement: jkac183_Supplementary_Fig_2 [file jkac183_supplementary_fig_2.pdf]

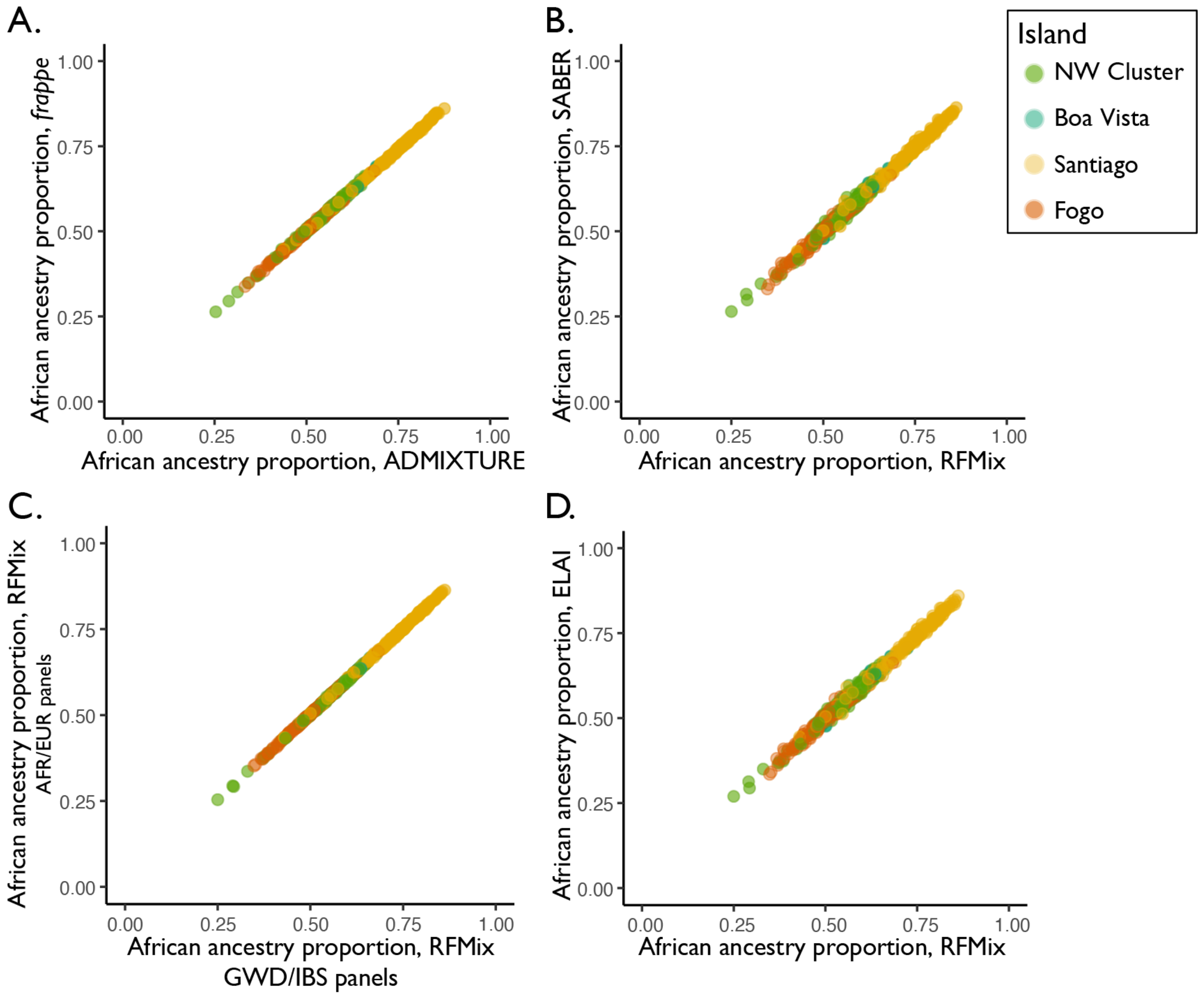

Supp Fig 2: Comparison to previous global and local ancestry inference. (A) ADMIXTURE estimates of overall autosomal ancestry per individual from this study are highly correlated (Pearson's $R > 0.99$, $p < 1 \times 10^{-8}$) with *frappe* estimates from Beleza et al. (2013). (B) Averaged autosomal ancestry per individual from local ancestry calls using RFMix are highly correlated (Pearson's $R > 0.99$, $p < 1 \times 10^{-8}$) with estimates from SABER local ancestry calls from Beleza et al. (2013). (C) RFMix local ancestry calling using 1kG resequenced genomes from all AFR and EUR populations correlates closely with RFMix local ancestry calling using GWD and IBS reference panels (Pearson's $R > 0.99$, $p < 1 \times 10^{-8}$). (D) Averaged autosomal ancestry per individual from local ancestry calls using RFMix correlate closely (Pearson's $R > 0.99$, $p < 1 \times 10^{-8}$) with those from ELAI.
