# Supplementary material for: Sex-biased admixture and assortative mating shape genetic variation and influence demographic inference in admixed Cabo Verdeans
Source: G3 (Bethesda). 2022 Jul 21;12(10):jkac183. doi: 10.1093/g3journal/jkac183 (PMC9526050; doi:10.1093/g3journal/jkac183)
Supplement: jkac183_Supplementary_Fig_3 [file jkac183_supplementary_fig_3.pdf]

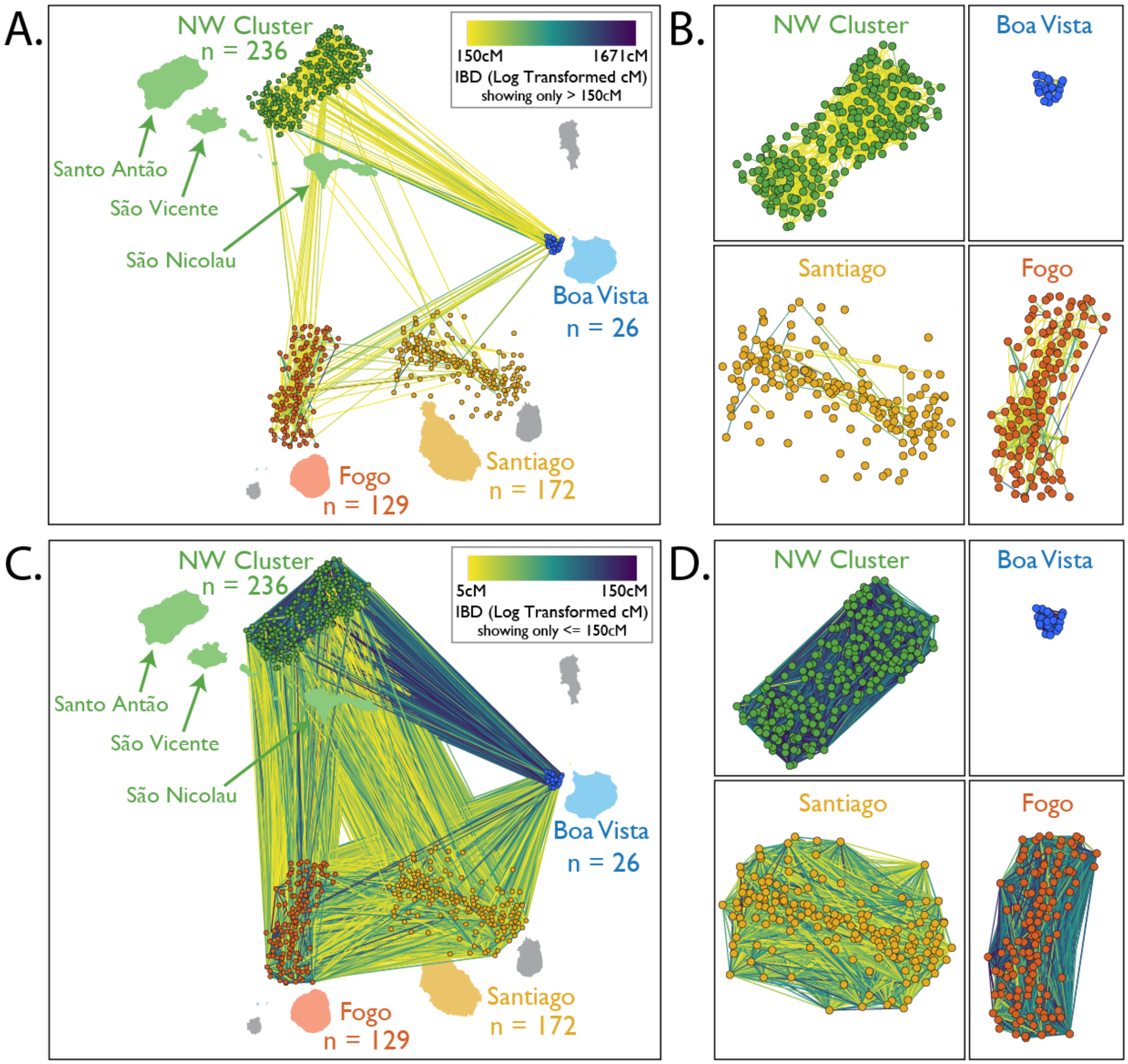

Supp Fig 3: Total IBD between and within the islands in the context of geography. The total length of segments identical-by-descent (IBD) are summed for each pairwise comparison of individuals. Here, we separately plot total IBD lengths over 150 cM (A and B) and less than or equal to 150 cM (C and D). In (A) and (C), each island has a corresponding cluster of nodes representing all sampled individuals. The edges between the nodes represent total IBD tract length shared between a pair of individuals. Individuals are localized to be adjacent to the island where they were sampled, and edges within an island's cluster attract nodes proportionally to shared IBD. Node placement within islands by a force-directed algorithm means that the spread of each cluster reflects the level of relatedness in each population. (B) and (D) show only the within-island IBD of (A) and (C), respectively. Note that (A) is the same as Figure 1, shown here for ease of comparison to within-island IBD.
