# Supplementary material for: Sex-biased admixture and assortative mating shape genetic variation and influence demographic inference in admixed Cabo Verdeans
Source: G3 (Bethesda). 2022 Jul 21;12(10):jkac183. doi: 10.1093/g3journal/jkac183 (PMC9526050; doi:10.1093/g3journal/jkac183)
Supplement: jkac183_Supplementary_Fig_4 [file jkac183_supplementary_fig_4.pdf]

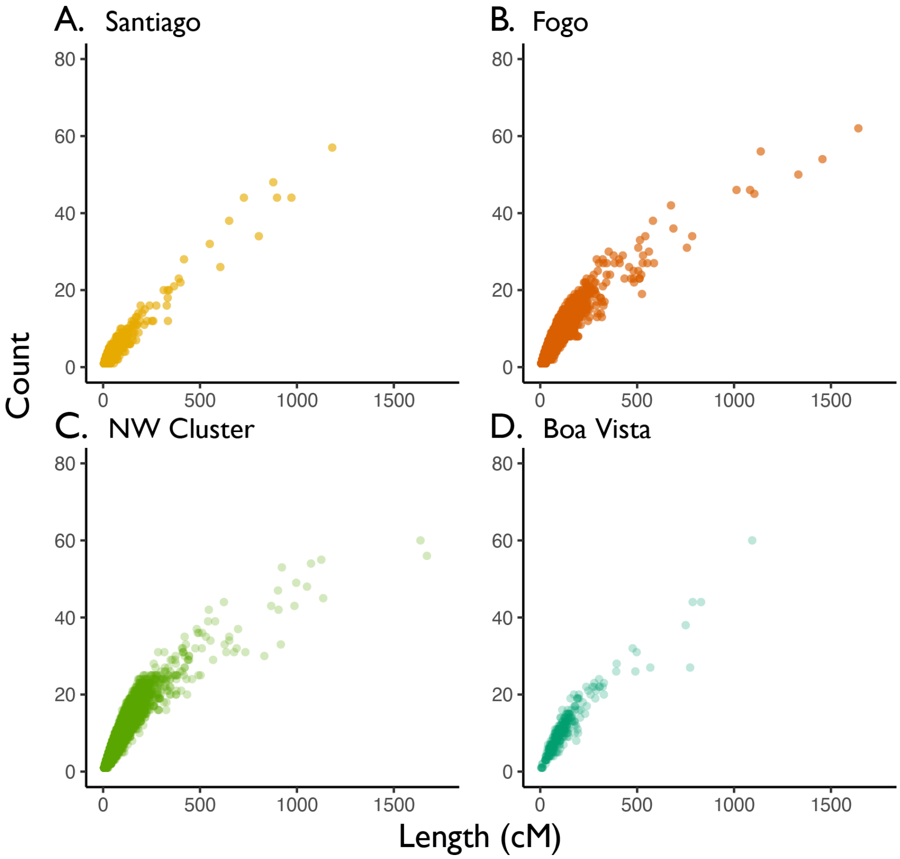

Supp Fig 4: IBD by island. Pairwise IBD sharing within islands. The total count and summed length of pairwise inferred IBD segments between individuals within each of four Cabo Verdean islands.
