# Supplementary material for: Sex-biased admixture and assortative mating shape genetic variation and influence demographic inference in admixed Cabo Verdeans
Source: G3 (Bethesda). 2022 Jul 21;12(10):jkac183. doi: 10.1093/g3journal/jkac183 (PMC9526050; doi:10.1093/g3journal/jkac183)
Supplement: jkac183_Supplementary_Fig_5 [file jkac183_supplementary_fig_5.pdf]

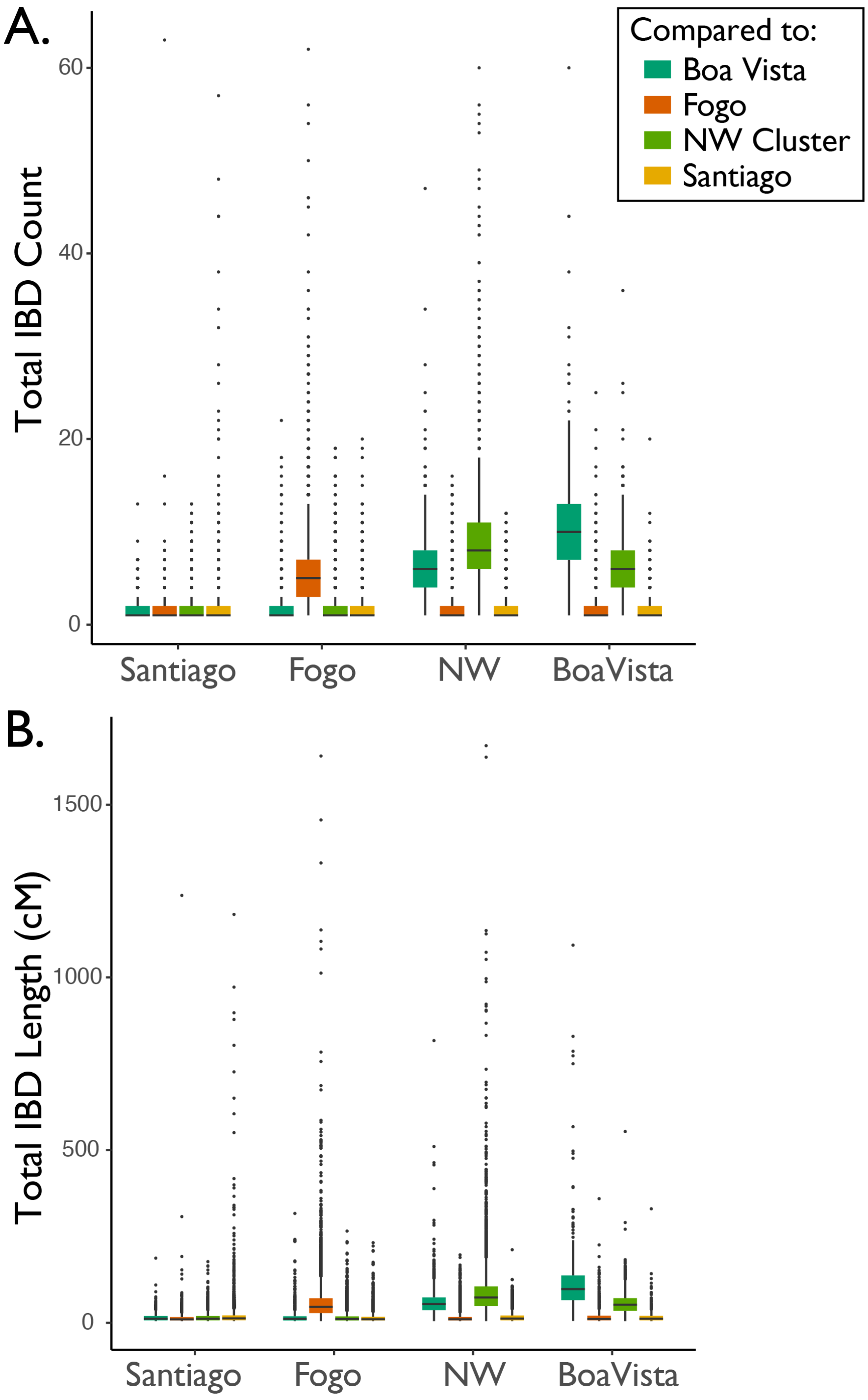

Supp Fig 5: The distribution of IBD sharing within and between islands. The distributions of total count (A) and summed length (B) of pairwise IBD segments shared between individuals both within the same island and across islands.
