# Supplementary material for: Sex-biased admixture and assortative mating shape genetic variation and influence demographic inference in admixed Cabo Verdeans
Source: G3 (Bethesda). 2022 Jul 21;12(10):jkac183. doi: 10.1093/g3journal/jkac183 (PMC9526050; doi:10.1093/g3journal/jkac183)
Supplement: jkac183_Supplementary_Fig_6 [file jkac183_supplementary_fig_6.pdf]

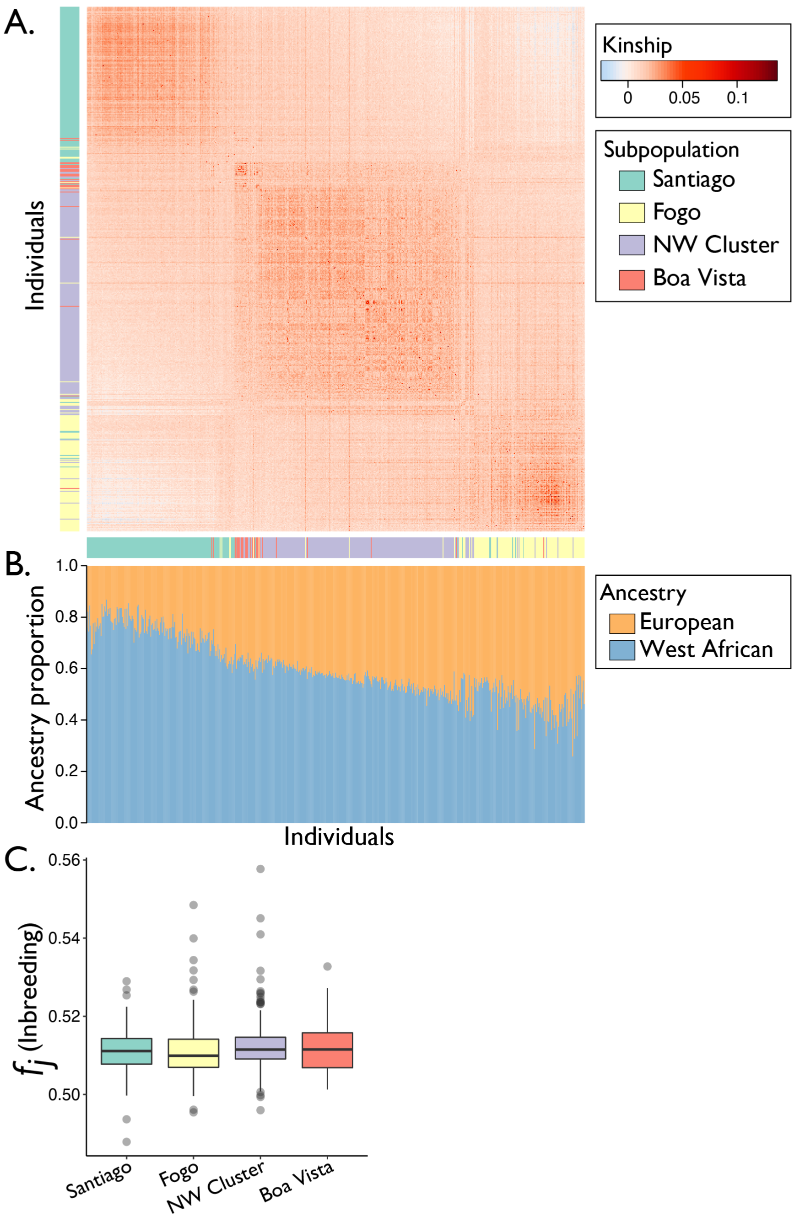

Supp Fig 6: Kinship estimation in the context of ancestry. Heatmap (A) showing the full kinship matrix and global ancestry estimates from ADMIXTURE (B) with individuals aligned with the kinship matrix, produced using the method of Ochoa & Storey (2019). Individuals within the matrix were ordered using seriation, which places low kinship values away from the diagonal. The diagonal of the kinship matrix gives the distribution of inbreeding coefficients (C), or f_j as described in Ochoa & Storey (2019), within each island region.
