# Supplementary material for: Sex-biased admixture and assortative mating shape genetic variation and influence demographic inference in admixed Cabo Verdeans
Source: G3 (Bethesda). 2022 Jul 21;12(10):jkac183. doi: 10.1093/g3journal/jkac183 (PMC9526050; doi:10.1093/g3journal/jkac183)
Supplement: jkac183_Supplementary_Fig_7 [file jkac183_supplementary_fig_7.pdf]

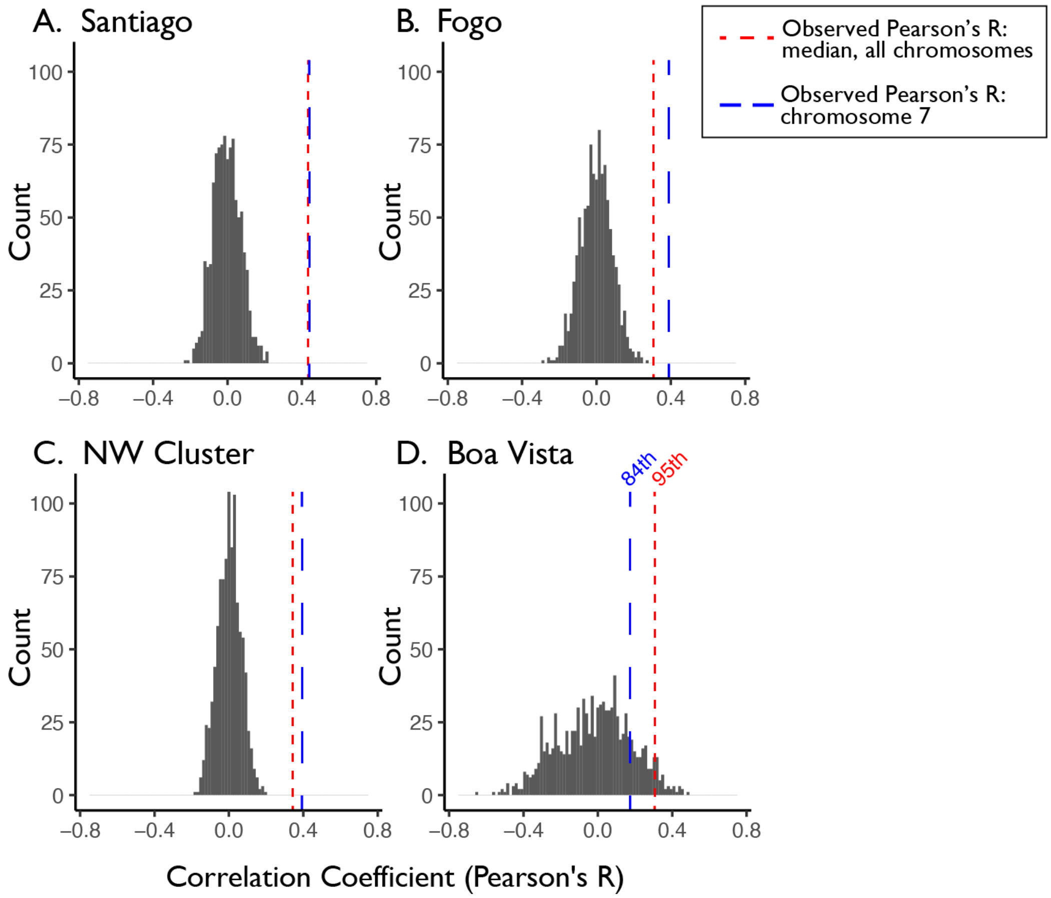

Supp Fig 7: Comparison of inferred ancestry-assortative mating strength to the sampling distribution based on randomly paired haplotypes. We compared the empirically-inferred strength of ancestry-assortative mating (red lines above: using the median Pearson's R from the set of all chromosomes shown in Fig 2B) to the correlation observed in random samples. Each distribution includes 1,000 sets of mating pairs that were randomly sampled with replacement from the full set of observed haplotypes in the current generation (using a representative chromosome, chromosome 7). The randomly sampled sets contained the same number of mating pairs as the empirical sample for each island (i.e., 172 pairs per sample for Santiago, 129 for Fogo, 236 for the NW Cluster, and 26 for Boa Vista). For all islands, the empirically-inferred strength of assortative mating differs significantly from the distribution of random samples (Santiago t-test: $t = 176.18$, $df = 998$, $p < 1 \times 10^{-8}$; Fogo t-test: $t = 113.75$, $df = 998$, $p < 1 \times 10^{-8}$; NW Cluster t-test: $t = 171.5$, $df = 998$, $p < 1 \times 10^{-8}$; Boa Vista t-test: $t = 52.07$, $df = 998$, $p < 1 \times 10^{-8}$; significance testing performed using the distribution of differences between empirical Pearson's R (red lines) and Pearson's R in each random sample to test whether the mean of that distribution differs from zero). The empirically-inferred strength of ancestry-assortative mating from a single example chromosome (chromosome 7) is also shown for comparison (blue lines).
