# Supplementary material for: Sex-biased admixture and assortative mating shape genetic variation and influence demographic inference in admixed Cabo Verdeans
Source: G3 (Bethesda). 2022 Jul 21;12(10):jkac183. doi: 10.1093/g3journal/jkac183 (PMC9526050; doi:10.1093/g3journal/jkac183)
Supplement: jkac183_Supplementary_Fig_8 [file jkac183_supplementary_fig_8.pdf]

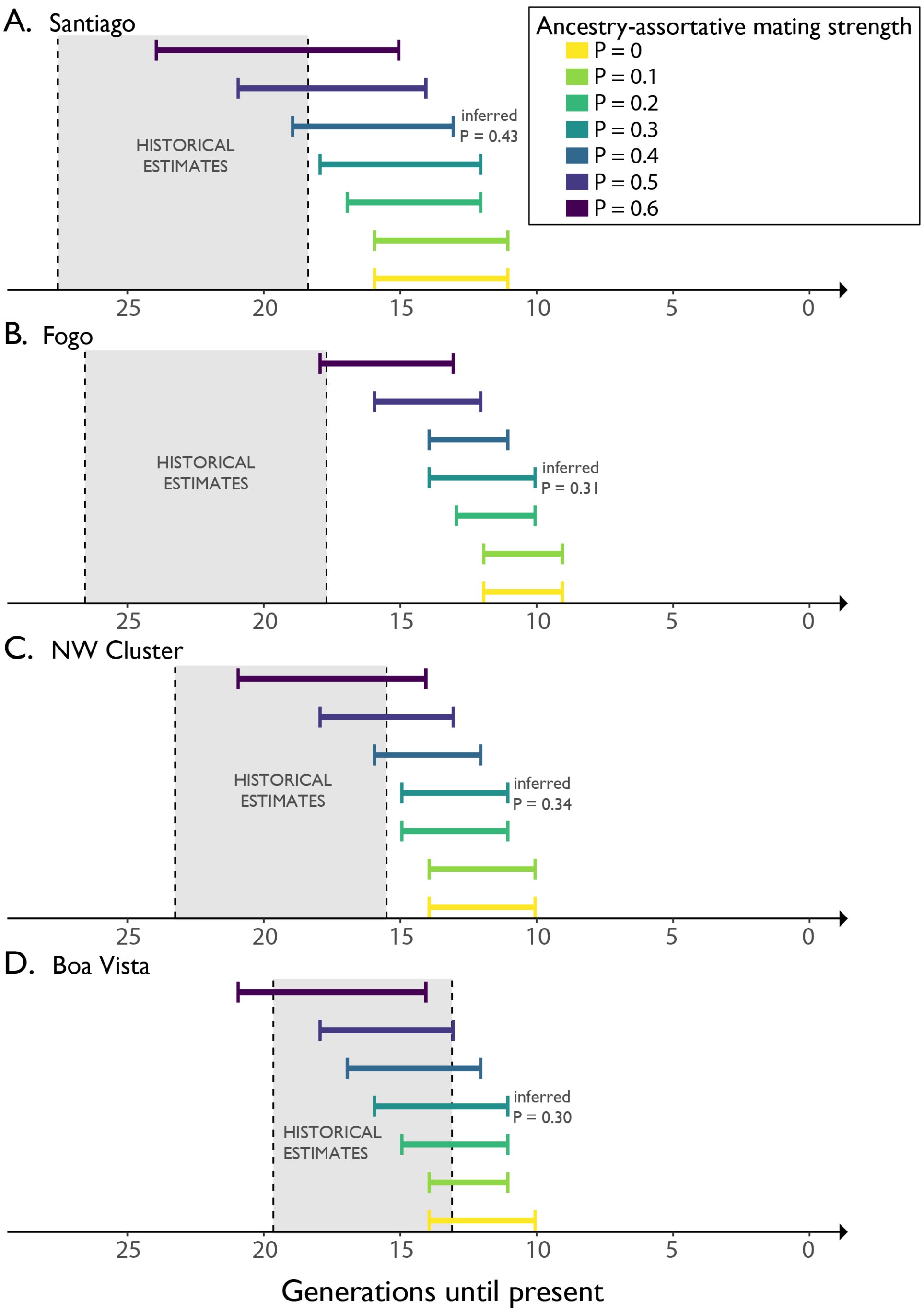

Supp Fig 8: Timelines of inferred generations of admixture using LAD under varying degrees of ancestry-assortative mating. For each island, admixture timing estimates inferred with the LAD-based method of Zaitlen et al. (2017) under a range of assortative mating strengths (P = the correlation in ancestries of individuals in mating pairs) are shown in comparison to historical records. Each result includes the range of estimates generated under the assumption of constant migration rate (m = 0.01; yielding older estimates) to the assumption of no migration (m = 0; yielding more recent estimates). For each island, the inferred value of P from Fig 2 is provided as an annotation to the right of the timing estimate that most closely corresponds to the inferred value.
