# Supplementary material for: Sex-biased admixture and assortative mating shape genetic variation and influence demographic inference in admixed Cabo Verdeans
Source: G3 (Bethesda). 2022 Jul 21;12(10):jkac183. doi: 10.1093/g3journal/jkac183 (PMC9526050; doi:10.1093/g3journal/jkac183)
Supplement: jkac183_Supplementary_Fig_9 [file jkac183_supplementary_fig_9.pdf]

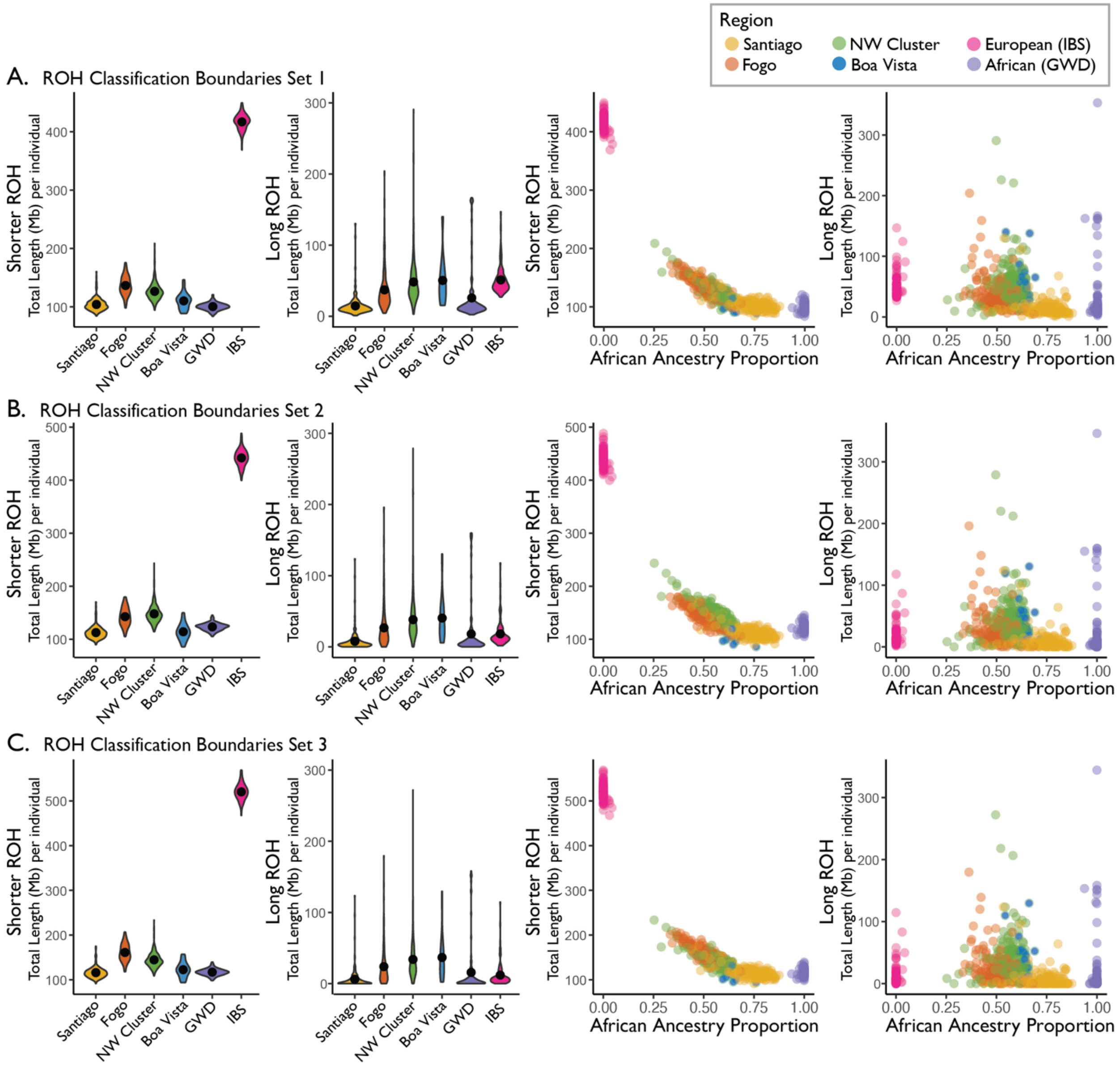

Supp Fig 9: ROH distributions under various length classification cutoffs. Repeating Fig 4 under three different sets of ROH length classification rules, the violin plots show the population-specific distributions of the total (summed over each genome) length of autosomal ROH per individual. The scatter plots show the total length of autosomal ROH per individual plotted against West African ancestry proportions and colored by population. (A) Set 1 uses the minimum shorter/long boundary (896,699 bp) reported in Pemberton et al. (2012), (B) Set 2 uses the mean shorter/long boundary (1,548,382 bp) reported in Pemberton et al. (2012), and (C) Set 3 uses the maximum shorter/long boundary (2,191,781 bp) reported in Pemberton et al. (2012).
