# Supplementary material for: Sex-biased admixture and assortative mating shape genetic variation and influence demographic inference in admixed Cabo Verdeans
Source: G3 (Bethesda). 2022 Jul 21;12(10):jkac183. doi: 10.1093/g3journal/jkac183 (PMC9526050; doi:10.1093/g3journal/jkac183)
Supplement: jkac183_Supplementary_Fig_10 [file jkac183_supplementary_fig_10.pdf]

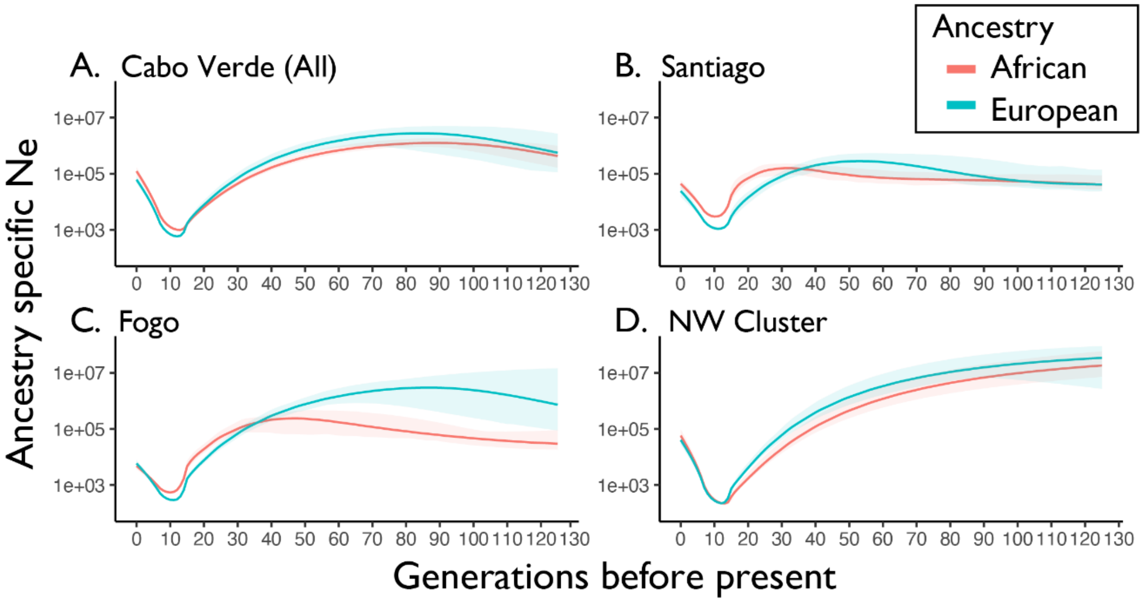

Supp Fig 10: Ancestry-specific population size. The estimated effective population sizes (Ne, plotted on a log scale) of West African and European ancestry plotted over time (generations until present). The solid lines show estimated ancestry-specific effective population sizes, and the shaded regions around the lines show 95% bootstrap confidence intervals.
