# Supplementary material for: Sex-biased admixture and assortative mating shape genetic variation and influence demographic inference in admixed Cabo Verdeans
Source: G3 (Bethesda). 2022 Jul 21;12(10):jkac183. doi: 10.1093/g3journal/jkac183 (PMC9526050; doi:10.1093/g3journal/jkac183)
Supplement: jkac183_Supplementary_Fig_11 [file jkac183_supplementary_fig_11.pdf]

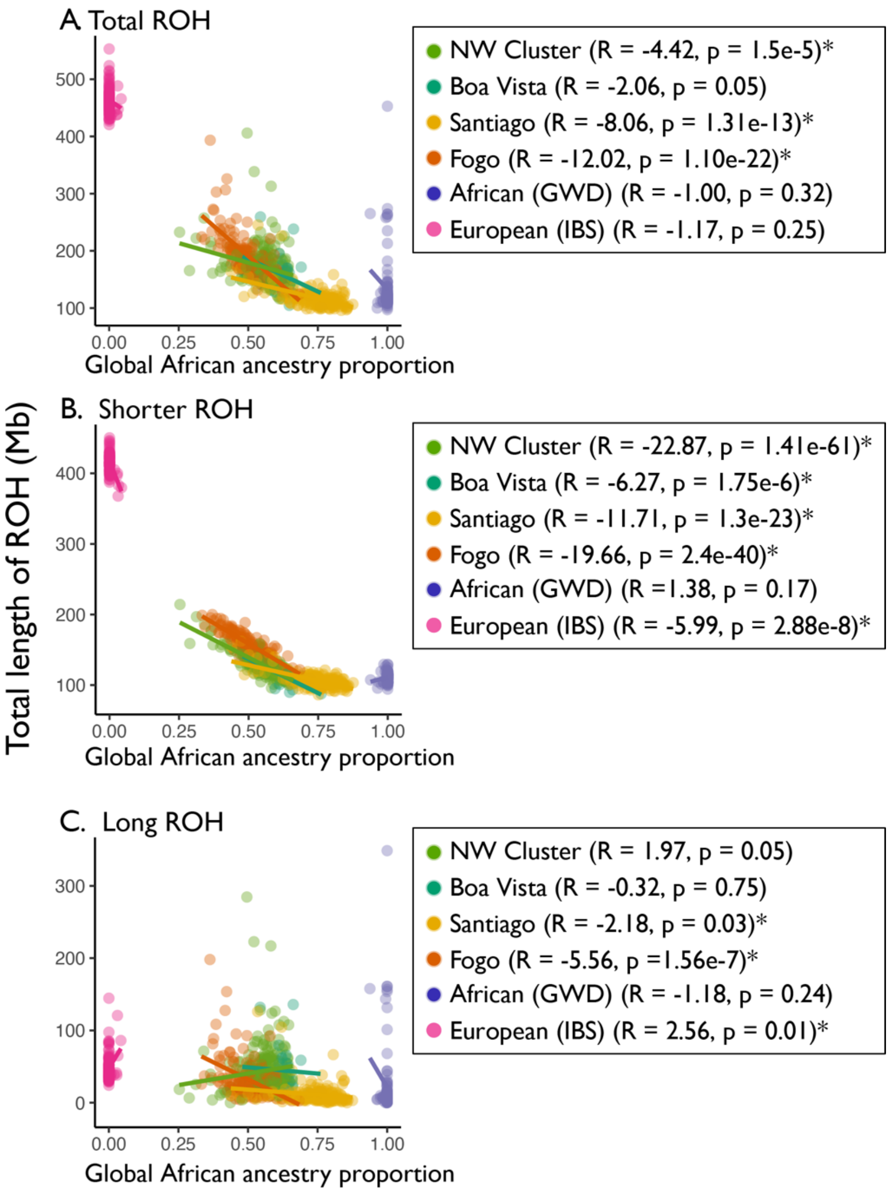

Supp Fig 11: ROH vs African ancestry proportion. For all ROH length classes (A), shorter ROH (B), and long ROH (C), the total length of autosomal ROH per individual is plotted against West African ancestry proportions and colored by population. For each length class, ROH within each population is regressed onto global African ancestry proportion.
