# Supplementary material for: Sex-biased admixture and assortative mating shape genetic variation and influence demographic inference in admixed Cabo Verdeans
Source: G3 (Bethesda). 2022 Jul 21;12(10):jkac183. doi: 10.1093/g3journal/jkac183 (PMC9526050; doi:10.1093/g3journal/jkac183)
Supplement: jkac183_Supplementary_Fig_12 [file jkac183_supplementary_fig_12.pdf]

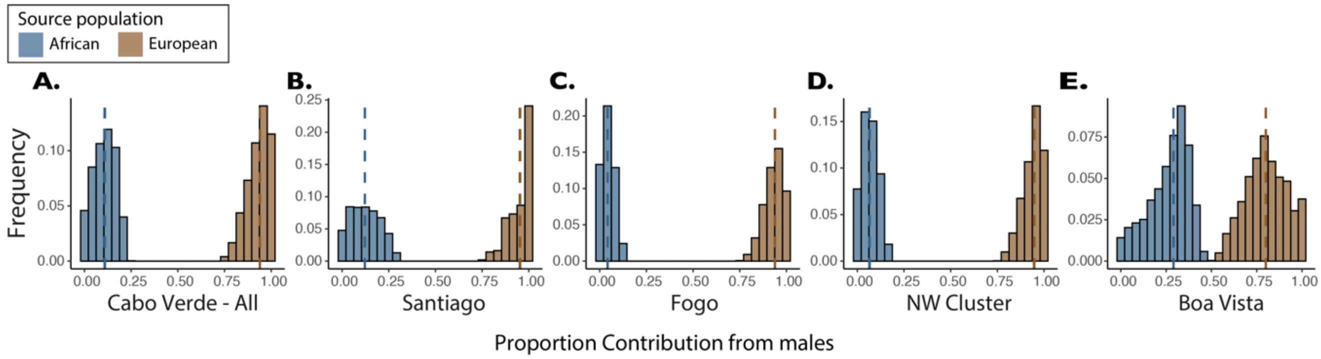

Supp Fig 12: Sex-biased admixture in Cabo Verde – male contributions. Under a model of constant admixture over time, the fraction of the total contribution of genetic material originating from males for West African and European source populations. Here, we show the distribution of parameter sets for the smallest 0.1% of Euclidean distances between the model-predicted and observed X and autosomal ancestry from a grid of possible parameter values. The range of sex-specific contributions from West African and European source populations that produce ancestry estimates closest to those observed in Cabo Verde are shown for Cabo Verde as a whole (A), and then broken down by region (B-E), with medians (dashed lines).
