# Supplementary material for: Sex-biased admixture and assortative mating shape genetic variation and influence demographic inference in admixed Cabo Verdeans
Source: G3 (Bethesda). 2022 Jul 21;12(10):jkac183. doi: 10.1093/g3journal/jkac183 (PMC9526050; doi:10.1093/g3journal/jkac183)
Supplement: jkac183_Supplementary_Table_1 [file jkac183_supplementary_table_1.pdf]

Supp Table 1: Summary of computational methods.

Program	Usage in this study	Reference
Characterization of ancestry		
PLINK v1.9	LD pruning; PCA	Purcell et al. 2007
ADMIXTURE v1.3.0	Unsupervised estimation of genomic ancestries	Alexander et al. 2009
SHAPEIT v2	Phasing	Delaneau et al. 2013
RFMix v1.5.4	Local ancestry calling	Maples et al. 2013
Inference of admixture timing		
ALDER v1.03	Estimation of admixture timing based on the extent of LD decay among neighboring loci	Loh et al. 2013
MULTIWAVER v2.0	Estimation of admixture timing based on ancestry tracts inferred by RFMix	Ni et al. 2019
LAD-based method	Estimation of admixture timing based on local ancestry disequilibrium (LAD)	Zaitlen et al. 2017
Testing for assortative mating and sex-biased admixture		
ANCESTOR	Estimation of the ancestry proportions of the two parents of each individual	Zou et al. 2015
Mechanistic model of sex-biased admixture	Inference of admixture parameters under a model allowing sex-biased contributions from source populations	Goldberg et al. 2015
IBD, ROH, and relatedness analyses		
Ancestry specific IBD Ne (ibdne.23Apr20.ae9.jar)	Estimation of ancestry-specific population sizes	Browning & Browning 2018
RefinedIBD (refined-ibd.17Jan20.102.jar)	Inference of segments of IBD	Browning & Browning 2015
Popkin	Kinship estimation under a framework designed for arbitrary population structure	Ochoa & Storey 2019
GARLIC v1.1.6	Classification of ROH	Szpiech et al. 2017
