# Supplementary material for: Sex-biased admixture and assortative mating shape genetic variation and influence demographic inference in admixed Cabo Verdeans
Source: G3 (Bethesda). 2022 Jul 21;12(10):jkac183. doi: 10.1093/g3journal/jkac183 (PMC9526050; doi:10.1093/g3journal/jkac183)
Supplement: jkac183_Supplementary_Table_2 [file jkac183_supplementary_table_2.pdf]

Supp Table 2: ROH length classification and LOD score cutoffs from Garlic.

Population	Class A/B Length Boundary	Class B/C Length Boundary	Window Size	LOD Score Cutoff
Santiago	303,824 bp	1,081,400 bp	50	2.203
NW Cluster	308,994 bp	1,076,210 bp	50	1.746
Fogo	309,213 bp	1,118,290 bp	50	0
Boa Vista	328,023 bp	1,144,080 bp	50	1.644
African (GWD)	327,265 bp	1,262,910 bp	50	1.773
European (IBS)	264,625 bp	914,139 bp	40	-2.169
