# Supplementary material for: Sex-biased admixture and assortative mating shape genetic variation and influence demographic inference in admixed Cabo Verdeans
Source: G3 (Bethesda). 2022 Jul 21;12(10):jkac183. doi: 10.1093/g3journal/jkac183 (PMC9526050; doi:10.1093/g3journal/jkac183)
Supplement: jkac183_Supplementary_Table_3 [file jkac183_supplementary_table_3.pdf]

**Supp Table 3: Estimates of admixture timing.**

| Population | Recorded generations[1] | Inferred generations: ALDER | Inferred generations: MultiWaver | Inferred generations: LAD, random mating | Inferred generations: LAD, assortative mating |
|---|---|---|---|---|---|
| Santiago | 18.37 – 27.55 | 8.81 - 10.05 | 6 – 9; 13 – 15 | 11 – 16 | 13 – 20 |
| NW Cluster | 15.5 – 23.25 | 10.33 – 11.53 | 7 – 10; 13 – 14 | 10 – 14 | 11 – 16 |
| Fogo | 17.7 – 26.55 | 9.74 – 10.52 | 11.5 – 12.5 | 9 – 12 | 10 – 13 |
| Boa Vista | 13.1 – 19.65 | 8.16 – 10.04 | 12 - 13 | 10 - 14 | 11 – 16 |

[1] See *Methods: Historical records* for the sources of historical estimates of admixture timing.
